# Supplementary material for: Resolving the full spectrum of human genome variation using Linked-Reads
Source: Genome Res. 2019 Apr;29(4):635–45. doi: 10.1101/gr.234443.118 (PMC6442396; doi:10.1101/gr.234443.118)
Supplement: Supplemental Material [file supp_gr.234443.118_Supplemental_longranger-2.2.zip › longranger-2.2.tar.bz2!/lr2.2-public/lib/go/src/code.google.com/vitess/docs/user-guide/topology-service.html]

Vitess / Topology Service


Toggle navigation

- Overview
- Guides
- Reference
- Blog
- GitHub

- #### Overview

  - What is Vitess
  - Scaling MySQL with Vitess
  - Key Concepts
- #### Getting Started

  - Run Vitess on Kubernetes
    - Custom Docker Build
  - Run Vitess Locally
- #### User Guide

  - Introduction- Client Libraries- Backing Up Data- Reparenting
        - Schema Management
        - VSchema Guide
        - Schema Swap (Tutorial)
        - Sharding
          - Horizontal Sharding (Tutorial, automated)
          - Horizontal Sharding (Tutorial, manual)
          - Sharding in Kubernetes (Tutorial, automated)
          - Sharding in Kubernetes (Tutorial, manual)
        - Vitess Sequences
        - MySQL Server Protocol
        - Vitess and Replication
        - Update Stream
        - Row Based Replication
        - Topology Service
        - Transport Security Model
        - Launching
          - Scalability Philosophy
          - Production Planning
          - Server Configuration
          - 2PC Guide
          - Troubleshooting
        - Upgrading
- #### Reference Guides

  - Vitess API- vtctl Commands
- #### Other Resources

  - Presentations- Blog- Roadmap
- #### Contributing

  - Contributing to Vitess- GitHub Workflow- Code Reviews
- GitHub

# Topology Service

- #### Overview

  - What is Vitess
  - Scaling MySQL with Vitess
  - Key Concepts
- #### Getting Started

  - Run Vitess on Kubernetes
    - Custom Docker Build
  - Run Vitess Locally
- #### User Guide

  - Introduction- Client Libraries- Backing Up Data- Reparenting
        - Schema Management
        - VSchema Guide
        - Schema Swap (Tutorial)
        - Sharding
          - Horizontal Sharding (Tutorial, automated)
          - Horizontal Sharding (Tutorial, manual)
          - Sharding in Kubernetes (Tutorial, automated)
          - Sharding in Kubernetes (Tutorial, manual)
        - Vitess Sequences
        - MySQL Server Protocol
        - Vitess and Replication
        - Update Stream
        - Row Based Replication
        - Topology Service
        - Transport Security Model
        - Launching
          - Scalability Philosophy
          - Production Planning
          - Server Configuration
          - 2PC Guide
          - Troubleshooting
        - Upgrading
- #### Reference Guides

  - Vitess API- vtctl Commands
- #### Other Resources

  - Presentations- Blog- Roadmap
- #### Contributing

  - Contributing to Vitess- GitHub Workflow- Code Reviews

# Topology Service

This document describes the Topology Service, a key part of the Vitess
architecture. This service is exposed to all Vitess processes, and is used to
store small pieces of configuration data about the Vitess cluster, and provide
cluster-wide locks. It also supports watches, which we will use soon.

Concretely, the Topology Service features are implemented by
a Lock Server, referred
to as Topology Server in the rest of this document. We use a plug-in
implementation and we support multiple Lock Servers (Zookeeper, etcd, Consul, …)
as backends for the service.

## Requirements and Usage

The Topology Service is used to store information about the Keyspaces, the
Shards, the Tablets, the Replication Graph, and the Serving Graph. We store
small data structures (a few hundred bytes) per object.

The main contract for the Topology Server is to be very highly available and
consistent. It is understood it will come at a higher latency cost and very low
throughput.

We never use the Topology Server as an RPC mechanism, nor as a storage system
for logs. We never depend on the Topology Server being responsive and fast to
serve every query.

The Topology Server must also support a Watch interface, to signal when certain
conditions occur on a node. This is used for instance to know when keyspaces
topology changes (for resharding for instance).

### Global vs Local

We differentiate two instances of the Topology Server: the Global instance, and
the per-cell Local instance:

- The Global instance is used to store global data about the topology that
  doesn’t change very often, for instance information about Keyspaces and
  Shards. The data is independent of individual instances and cells, and needs
  to survive a cell going down entirely.
- There is one Local instance per cell, that contains cell-specific information,
  and also rolled-up data from the global + local cell to make it easier for
  clients to find the data. The Vitess local processes should not use the Global
  topology instance, but instead the rolled-up data in the Local topology
  server as much as possible.

The Global instance can go down for a while and not impact the local cells (an
exception to that is if a reparent needs to be processed, it might not work). If
a Local instance goes down, it only affects the local tablets in that instance
(and then the cell is usually in bad shape, and should not be used).

Furthermore, the Vitess processes will not use the Global nor the Local Topology
Server to serve individual queries. They only use the Topology Server to get the
topology information at startup and in the background, but never to directly
serve queries.

### Recovery

If a local Topology Server dies and is not recoverable, it can be wiped out. All
the tablets in that cell then need to be restarted so they re-initialize their
topology records (but they won’t lose any MySQL data).

If the global Topology Server dies and is not recoverable, this is more of a
problem. All the Keyspace / Shard objects have to be re-created. Then the cells
should recover.

## Global Data

This section describes the data structures stored in the global instance of the
topology server.

### Keyspace

The Keyspace object contains various information, mostly about sharding: how is
this Keyspace sharded, what is the name of the sharding key column, is this
Keyspace serving data yet, how to split incoming queries, …

An entire Keyspace can be locked. We use this during resharding for instance,
when we change which Shard is serving what inside a Keyspace. That way we
guarantee only one operation changes the keyspace data concurrently.

### Shard

A Shard contains a subset of the data for a Keyspace. The Shard record in the
global topology contains:

- the MySQL Master tablet alias for this shard
- the sharding key range covered by this Shard inside the Keyspace
- the tablet types this Shard is serving (master, replica, batch, …), per cell
  if necessary.
- if during filtered replication, the source shards this shard is replicating
  from
- the list of cells that have tablets in this shard
- shard-global tablet controls, like blacklisted tables no tablet should serve
  in this shard

A Shard can be locked. We use this during operations that affect either the
Shard record, or multiple tablets within a Shard (like reparenting), so multiple
jobs don’t concurrently alter the data.

### VSchema Data

The VSchema data contains sharding and routing information for
the VTGate V3 API.

## Local Data

This section describes the data structures stored in the local instance (per
cell) of the topology server.

### Tablets

The Tablet record has a lot of information about a single vttablet process
running inside a tablet (along with the MySQL process):

- the Tablet Alias (cell+unique id) that uniquely identifies the Tablet
- the Hostname, IP address and port map of the Tablet
- the current Tablet type (master, replica, batch, spare, …)
- which Keyspace / Shard the tablet is part of
- the sharding Key Range served by this Tablet
- user-specified tag map (to store per installation data for instance)

A Tablet record is created before a tablet can be running (either by `vtctl
InitTablet` or by passing the `init_*` parameters to vttablet). The only way a
Tablet record will be updated is one of:

- The vttablet process itself owns the record while it is running, and can
  change it.
- At init time, before the tablet starts
- After shutdown, when the tablet gets deleted.
- If a tablet becomes unresponsive, it may be forced to spare to make it
  unhealthy when it restarts.

### Replication Graph

The Replication Graph allows us to find Tablets in a given Cell / Keyspace /
Shard. It used to contain information about which Tablet is replicating from
which other Tablet, but that was too complicated to maintain. Now it is just a
list of Tablets.

### Serving Graph

The Serving Graph is what the clients use to find the per-cell topology of a
Keyspace. It is a roll-up of global data (Keyspace + Shard). vtgates only open a
small number of these objects and get all they need quickly.

#### SrvKeyspace

It is the local representation of a Keyspace. It contains information on what
shard to use for getting to the data (but not information about each individual
shard):

- the partitions map is keyed by the tablet type (master, replica, batch, …) and
  the values are list of shards to use for serving.
- it also contains the global Keyspace fields, copied for fast access.

It can be rebuilt by running `vtctl RebuildKeyspaceGraph`. It is
automatically rebuilt when a tablet starts up in a cell and the SrvKeyspace
for that cell / keyspace doesn't exist yet. It will also be changed
during horizontal and vertical splits.

#### SrvVSchema

It is the local roll-up for the VSchema. It contains the VSchema for all
keyspaces in a single object.

It can be rebuilt by running `vtctl RebuildVSchemaGraph`. It is automatically
rebuilt when using `vtctl ApplyVSchema` (unless prevented by flags).

## Workflows Involving the Topology Server

The Topology Server is involved in many Vitess workflows.

When a Tablet is initialized, we create the Tablet record, and add the Tablet to
the Replication Graph. If it is the master for a Shard, we update the global
Shard record as well.

Administration tools need to find the tablets for a given Keyspace / Shard:
first we get the list of Cells that have Tablets for the Shard (global topology
Shard record has these) then we use the Replication Graph for that Cell /
Keyspace / Shard to find all the tablets then we can read each tablet record.

When a Shard is reparented, we need to update the global Shard record with the
new master alias.

Finding a tablet to serve the data is done in two stages: vtgate maintains a
health check connection to all possible tablets, and they report which keyspace
/ shard / tablet type they serve. vtgate also reads the SrvKeyspace object, to
find out the shard map. With these two pieces of information, vtgate can route
the query to the right vttablet.

During resharding events, we also change the topology a lot. An horizontal split
will change the global Shard records, and the local SrvKeyspace records. A
vertical split will change the global Keyspace records, and the local
SrvKeyspace records.

## Implementations

The Topology Server interface is defined in our code in `go/vt/topo/server.go`
and we also have a set of unit tests for it in `go/vt/topo/test`.

This part describes the two implementations we have, and their specific
behavior.

If starting from scratch, please use the `zk2`, `etcd2` or `consul`
implementations, as we are deprecating the old `zookeeper` and `etcd`
implementations. See the migration section below if you want to migrate.

### Zookeeper `zk2` Implementation (new version of `zookeeper`)

This is the recommended implementation when using Zookeeper. The old `zookeeper`
implementation is deprecated, see next section.

The global cell typically has around 5 servers, distributed one in each
cell. The local cells typically have 3 or 5 servers, in different server racks /
sub-networks for higher resilience. For our integration tests, we use a single
ZK server that serves both global and local cells.

We provide the `zk` utility for easy access to the topology data in
Zookeeper. It can list, read and write files inside any Zoopeeker server. Just
specify the `-server` parameter to point to the Zookeeper servers. Note the
vtctld UI can also be used to see the contents of the topology data.

To configure a Zookeeper installation, let's start with the global cell
service. It is described by the addresses of the servers (comma separated list),
and by the root directory to put the Vitess data in. For instance, assuming we
want to use servers `global_server1,global_server2` in path `/vitess/global`:

```
# First create the directory in the global server:
zk -server global_server1,global_server2 touch -p /vitess/global

# Set the following flags to let Vitess use this global server:
# -topo_implementation zk2
# -topo_global_server_address global_server1,global_server2
# -topo_global_root /vitess/global
```

Then to add a cell whose local topology servers `cell1_server1,cell1_server2`
will store their data under the directory `/vitess/cell1`:

```
TOPOLOGY="-topo_implementation zk2 -topo_global_server_address global_server1,global_server2 -topo_global_root /vitess/global"

# Reference cell1 in the global topology service:
vtctl $TOPOLOGY AddCellInfo \
  -server_address cell1_server1,cell1_server2 \
  -root /vitess/cell1 \
  cell1
```

If only one cell is used, the same Zookeeper instance can be used for both
global and local data. A local cell record still needs to be created, just use
the same server address, and very importantly a *different* root directory.

#### Implementation Details

We use the following paths:

*Global Cell*:

- Election path: `elections/<name>`
- CellInfo path: `cells/<cell name>/CellInfo`
- Keyspace: `keyspaces/<keyspace>/Keyspace`
- Shard: `keyspaces/<keyspace>/shards/<shard>/Shard`
- VSchema: `keyspaces/<keyspace>/VSchema`

*Local Cell*:

- Tablet: `tablets/<cell>-<uid>/Tablet`
- Replication Graph: `keyspaces/<keyspace>/shards/<shard>/ShardReplication`
- SrvKeyspace: `keyspaces/<keyspace>/SrvKeyspace`
- SrvVSchema: `SvrVSchema`

For locks, we create a subdirectory called `locks` under either the keyspace
directory or the shard directory.

Both locks and master election are implemented using ephemeral, sequential files
which are stored in their respective directory.

We store the proto3 binary data for each object. The `zk` utility can decode
these files when using the `-p` option of the `cat` command:

```
$ zk --server localhost:15014 cat -p /global/keyspaces/test_keyspace/shards/-80/Shard
master_alias: <
  cell: "test_nj"
  uid: 62344
>
key_range: <
  end: "\200"
>
served_types: <
  tablet_type: MASTER
>
served_types: <
  tablet_type: REPLICA
>
served_types: <
  tablet_type: RDONLY
>
cells: "test_nj"
```

### Old Zookeeper `zookeeper` Implementaion (deprecated, use `zk2` instead)

This old `zookeeper` topology service is deprecated, and will be removed in
Vitess version 2.2. Please use `zk2` instead, and see the `topo2topo` section
below for migration.

Our Zookeeper implementation is based on a configuration file that describes
where the global and each local cell ZK instances are. When adding a cell, all
processes that may access that cell should be restarted with the new
configuration file.

The global cell typically has around 5 servers, distributed one in each
cell. The local cells typically have 3 or 5 servers, in different server racks /
sub-networks for higher resilience. For our integration tests, we use a single
ZK server that serves both global and local cells.

We sometimes store both data and sub-directories in a path (for a keyspace for
instance). We use JSON to encode the data.

For locking, we use an auto-incrementing file name in the `/action` subdirectory
of the object directory. We also move them to `/actionlogs` when the lock is
released. And we have a purge process to clear the old locks (which should be
run on a crontab, typically).

Note the paths used to store global and per-cell data do not overlap, so a
single ZK can be used for both global and local ZKs. This is however not
recommended, for reliability reasons.

- Keyspace: `/zk/global/vt/keyspaces/<keyspace>`
- Shard: `/zk/global/vt/keyspaces/<keyspace>/shards/<shard>`
- Tablet: `/zk/<cell>/vt/tablets/<uid>`
- Replication Graph: `/zk/<cell>/vt/replication/<keyspace>/<shard>`
- SrvKeyspace: `/zk/<cell>/vt/ns/<keyspace>`
- SrvVSchema: `/zk/<cell>/vt/vschema`

We provide the 'zk' utility for easy access to the topology data in
Zookeeper. For instance:

```
# NOTE: We do not set the ZK_CLIENT_CONFIG environment variable here,
# as the zk tool connects to a specific server. 
$ zk -server <server address> ls /zk/global/vt/keyspaces/user
action
actionlog
shards
```

### etcd `etcd2` Implementation (new version of `etcd`)

This topology service plugin is meant to use etcd clusters as storage backend
for the topology data. This topology service supports version 3 and up of the
etcd server.

This implementation is named `etcd2` because it supersedes our previous
implementation `etcd`. Note that the storage format has been changed with the
`etcd2` implementation, i.e. existing data created by the previous `etcd`
implementation must be migrated manually (See migration section below).

To configure an `etcd2` installation, let's start with the global cell
service. It is described by the addresses of the servers (comma separated list),
and by the root directory to put the Vitess data in. For instance, assuming we
want to use servers `http://global_server1,http://global_server2` in path
`/vitess/global`:

```
# Set the following flags to let Vitess use this global server,
# and simplify the example below:
# -topo_implementation etcd2
# -topo_global_server_address http://global_server1,http://global_server2
# -topo_global_root /vitess/global
TOPOLOGY="-topo_implementation etcd2 -topo_global_server_address http://global_server1,http://global_server2 -topo_global_root /vitess/global
```

Then to add a cell whose local topology servers
`http://cell1_server1,http://cell1_server2` will store their data under the
directory `/vitess/cell1`:

```
# Reference cell1 in the global topology service:
# (the TOPOLOGY variable is defined in the previous section)
vtctl $TOPOLOGY AddCellInfo \
  -server_address http://cell1_server1,http://cell1_server2 \
  -root /vitess/cell1 \
  cell1
```

If only one cell is used, the same etcd instances can be used for both
global and local data. A local cell record still needs to be created, just use
the same server address and, very importantly, a *different* root directory.

#### Implementation Details

We use the following paths:

*Global Cell*:

- Election path: `elections/<name>`
- CellInfo path: `cells/<cell name>/CellInfo`
- Keyspace: `keyspaces/<keyspace>/Keyspace`
- Shard: `keyspaces/<keyspace>/shards/<shard>/Shard`
- VSchema: `keyspaces/<keyspace>/VSchema`

*Local Cell*:

- Tablet: `tablets/<cell>-<uid>/Tablet`
- Replication Graph: `keyspaces/<keyspace>/shards/<shard>/ShardReplication`
- SrvKeyspace: `keyspaces/<keyspace>/SrvKeyspace`
- SrvVSchema: `SvrVSchema`

For locks, we use a subdirectory named `locks` in the directory to lock, and an
ephemeral file in that subdirectory (it is associated with a lease, whose TTL
can be set with the `-topo_etcd_lease_duration` flag, defaults to 30
seconds). The ephemeral file with the lowest ModRevision has the lock, the
others wait for files with older ModRevisions to disappear.

Master elections also use a subdirectory, named after the election Name, and use
a similar method as the locks, with ephemeral files.

We store the proto3 binary data for each object (as the v3 API allows us to store binary data).

### Old etcd `etcd` Implementaion (deprecated, use `etcd2` instead)

This old `etcd` topology service is deprecated, and will be removed in
Vitess version 2.2. Please use `etcd2` instead, and see the `topo2topo` section
below for migration.

Our etcd implementation is based on a command-line parameter that gives the
location(s) of the global etcd server. Then we query the path `/vt/cells` and
each file in there is named after a cell, and contains the list of etcd servers
for that cell. Each cell server files are stored in `/vt/`.

We use the `_Data` filename to store the data, JSON encoded.

For locking, we store a `_Lock` file with various contents in the directory that
contains the object to lock.

We use the following paths:

- Keyspace: `/vt/keyspaces/<keyspace>/_Data`
- Shard: `/vt/keyspaces/<keyspace>/<shard>/_Data`
- Tablet: `/vt/tablets/<cell>-<uid>/_Data`
- Replication Graph: `/vt/replication/<keyspace>/<shard>/_Data`
- SrvKeyspace: `/vt/ns/<keyspace>/_Data`
- SrvVSchema: `/vt/ns/_VSchema`

### Consul `consul` Implementation

This topology service plugin is meant to use Consul clusters as storage backend
for the topology data.

To configure a `consul` installation, let's start with the global cell
service. It is described by the address of a server,
and by the root node path to put the Vitess data in (it cannot start with `/`). For instance, assuming we
want to use servers `global_server:global_port` with node path
`vitess/global`:

```
# Set the following flags to let Vitess use this global server,
# and simplify the example below:
# -topo_implementation consul
# -topo_global_server_address global_server:global_port
# -topo_global_root vitess/global
TOPOLOGY="-topo_implementation consul -topo_global_server_address global_server:global_port -topo_global_root vitess/global
```

Then to add a cell whose local topology server
`cell1_server1:cell1_port` will store their data under the
directory `vitess/cell1`:

```
# Reference cell1 in the global topology service:
# (the TOPOLOGY variable is defined in the previous section)
vtctl $TOPOLOGY AddCellInfo \
  -server_address cell1_server1:cell1_port \
  -root vitess/cell1 \
  cell1
```

If only one cell is used, the same consul instances can be used for both
global and local data. A local cell record still needs to be created, just use
the same server address and, very importantly, a *different* root node path.

#### Implementation Details

We use the following paths:

*Global Cell*:

- Election path: `elections/<name>`
- CellInfo path: `cells/<cell name>/CellInfo`
- Keyspace: `keyspaces/<keyspace>/Keyspace`
- Shard: `keyspaces/<keyspace>/shards/<shard>/Shard`
- VSchema: `keyspaces/<keyspace>/VSchema`

*Local Cell*:

- Tablet: `tablets/<cell>-<uid>/Tablet`
- Replication Graph: `keyspaces/<keyspace>/shards/<shard>/ShardReplication`
- SrvKeyspace: `keyspaces/<keyspace>/SrvKeyspace`
- SrvVSchema: `SvrVSchema`

For locks, we use a file named `Lock` in the directory to lock, and the regular
Consul Lock API.

Master elections use a single lock file (the Election path) and the regular
Consul Lock API. The contents of the lock file is the ID of the current master.

Watches use the Consul long polling Get call. They cannot be interrupted, so we
use a long poll whose duration is set by the `-topo_consul_watch_poll_duration`
flag. Canceling a watch may have to wait until the end of a polling cycle with
that duration before returning.

We store the proto3 binary data for each object.

## Running In Only One Cell

The topology service is meant to be distributed across multiple cells, and
survive single cell outages. However, one common usage is to run a Vitess
cluster in only one cell / region. This part explains how to do this, and later
on upgrade to multiple cells / regions.

If running in a single cell, the same topology service can be used for both
global and local data. A local cell record still needs to be created, just use
the same server address and, very importantly, a *different* root node path.

In that case, just running 3 servers for topology service quorum is probably
sufficient. For instance, 3 etcd servers. And use their address for the local
cell as well. Let's use a short cell name, like `local`, as the local data in
that topology server will later on be moved to a different topology service,
which will have the real cell name.

### Extending To More Cells

To then run in multiple cells, the current topology service needs to be split
into a global instance and one local instance per cell. Whereas, the initial
setup had 3 topology servers (used for global and local data), we recommend to
run 5 global servers across all cells (for global topology data) and 3 local
servers per cell (for per-cell topology data).

To migrate to such a setup, start by adding the 3 local servers in the second
cell and run `vtctl AddCellinfo` as was done for the first cell. Tablets and
vtgates can now be started in the second cell, and used normally.

vtgate can then be configured with a list of cells to watch for tablets using
the `-cells_to_watch` command line parameter. It can then use all tablets in all
cells to route traffic. Note this is necessary to access the master in another
cell.

After the extension to two cells, the original topo service contains both the
global topology data, and the first cell topology data. The more symetrical
configuration we're after would be to split that original service into two: a
global one that only contains the global data (spread across both cells), and a
local one to the original cells. To achieve that split:

- Start up a new local topology service in that original cell (3 more local
  servers in that cell).
- Pick a name for that cell, different from `local`.
- Use `vtctl AddCellInfo` to configure it.
- Make sure all vtgates can see that new local cell (again, using
  `-cells_to_watch`).
- Restart all vttablets to be in that new cell, instead of the `local` cell name
  used before.
- Use `vtctl RemoveKeyspaceCell` to remove all mentions of the `local` cell in
  all keyspaces.
- Use `vtctl RemoveCellInfo` to remove the global configurations for that
  `local` cell.
- Remove all remaining data in the global topology service that are in the old
  local server root.

After this split, the configuration is completely symetrical:

- a global topology service, with servers in all cells. Only contains global
  topology data about Keyspaces, Shards and VSchema. Typically it has 5 servers
  across all cells.
- a local topology service to each cell, with servers only in that cell. Only
  contains local topology data about Tablets, and roll-ups of global data for
  efficient access. Typically, it has 3 servers in each cell.

## Migration Between Implementations

We provide the `topo2topo` binary file to migrate between one implementation
and another of the topology service.

The process to follow in that case is:

- Start from a stable topology, where no resharding or reparenting is on-going.
- Configure the new topology service so it has at least all the cells of the
  source topology service. Make sure it is running.
- Run the `topo2topo` program with the right flags. `-from_implementation`,
  `-from_root`, `-from_server` describe the source (old) topology
  service. `-to_implementation`, `-to_root`, `-to_server` describe the
  destination (new) topology service.
- Run `vtctl RebuildKeyspaceGraph` for each keyspace using the new topology
  service flags.
- Run `vtctl RebuildVSchemaGraph` using the new topology service flags.
- Restart all `vtgate` using the new topology service flags. They will see the
  same keyspaces / shards / tablets / vschema as before, as the topology was
  copied over.
- Restart all `vttablet` using the new topology service flags. They may use the
  same ports or not, but they will update the new topology when they start up,
  and be visible from vtgate.
- Restart all `vtctld` processes using the new topology service flags. So that
  the UI also shows the new data.

Sample commands to migrate from deprecated `zookeeper` to `zk2`
topology would be:

```
# Let's assume the zookeeper client config file is already
# exported in $ZK_CLIENT_CONFIG, and it contains a global record
# pointing to: global_server1,global_server2
# an a local cell cell1 pointing to cell1_server1,cell1_server2
#
# The existing directories created by Vitess are:
# /zk/global/vt/...
# /zk/cell1/vt/...
#
# The new zk2 implementation can use any root, so we will use:
# /vitess/global in the global topology service, and:
# /vitess/cell1 in the local topology service.

# Create the new topology service roots in global and local cell.
zk -server global_server1,global_server2 touch -p /vitess/global
zk -server cell1_server1,cell1_server2 touch -p /vitess/cell1

# Store the flags in a shell variable to simplify the example below.
TOPOLOGY="-topo_implementation zk2 -topo_global_server_address global_server1,global_server2 -topo_global_root /vitess/global"

# Reference cell1 in the global topology service:
vtctl $TOPOLOGY AddCellInfo \
  -server_address cell1_server1,cell1_server2 \
  -root /vitess/cell1 \
  cell1

# Now copy the topology. Note the old zookeeper implementation doesn't need
# any server or root parameter, as it reads ZK_CLIENT_CONFIG.
topo2topo \
  -from_implementation zookeeper \
  -to_implementation zk2 \
  -to_server global_server1,global_server2 \
  -to_root /vitess/global \

# Rebuild SvrKeyspace objects in new service, for each keyspace.
vtctl $TOPOLOGY RebuildKeyspaceGraph keyspace1
vtctl $TOPOLOGY RebuildKeyspaceGraph keyspace2

# Rebuild SrvVSchema objects in new service.
vtctl $TOPOLOGY RebuildVSchemaGraph

# Now restart all vtgate, vttablet, vtctld processes replacing:
# -topo_implementation zookeeper
# With:
# -topo_implementation zk2
# -topo_global_server_address global_server1,global_server2
# -topo_global_root /vitess/global
#
# After this, the ZK_CLIENT_CONF file and environment variables are not needed
# any more.
```

Contact: vitess@googlegroups.com  **·**  
Announcements  **·**  
© 2017 Vitess powered by Google Inc
